# Genome-Wide Analysis in Brazilian Xavante Indians Reveals Low Degree of Admixture

**DOI:** 10.1371/journal.pone.0042702

**Published:** 2012-08-10

**Authors:** Patricia C. Kuhn, Andréa R. V. Russo. Horimoto, José Maurício Sanches, João Paulo B. Vieira Filho, Luciana Franco, Amaury Dal Fabbro, Laercio Joel Franco, Alexandre C. Pereira, Regina S Moises

**Affiliations:** 1 Disciplina de Endocrinologia, Escola Paulista de Medicina, Universidade Federal de São Paulo, São Paulo, Brazil; 2 Laboratory of Genetics and Molecular Cardiology, Heart Institute, Medical School of University of São Paulo, São Paulo, Brazil; 3 Departamento de Medicina Social, Faculdade de Medicina de Ribeirão Preto, Universidade de São Paulo, Ribeirão Preto, Brazil; University of Utah, United States of America

## Abstract

Characterization of population genetic variation and structure can be used as tools for research in human genetics and population isolates are of great interest. The aim of the present study was to characterize the genetic structure of Xavante Indians and compare it with other populations. The Xavante, an indigenous population living in Brazilian Central Plateau, is one of the largest native groups in Brazil. A subset of 53 unrelated subjects was selected from the initial sample of 300 Xavante Indians. Using 86,197 markers, Xavante were compared with all populations of HapMap Phase III and HGDP-CEPH projects and with a Southeast Brazilian population sample to establish its population structure. Principal Components Analysis showed that the Xavante Indians are concentrated in the Amerindian axis near other populations of known Amerindian ancestry such as Karitiana, Pima, Surui and Maya and a low degree of genetic admixture was observed. This is consistent with the historical records of bottlenecks experience and cultural isolation. By calculating pair-wise F_st_ statistics we characterized the genetic differentiation between Xavante Indians and representative populations of the HapMap and from HGDP-CEPH project. We found that the genetic differentiation between Xavante Indians and populations of Ameridian, Asian, European, and African ancestry increased progressively. Our results indicate that the Xavante is a population that remained genetically isolated over the past decades and can offer advantages for genome-wide mapping studies of inherited disorders.

## Introduction

Knowledge of genetic diversity patterns of human populations provides important insights of their evolutionary history and is useful in genetic mapping studies of complex diseases and their component traits [1]–[3]. In this context, isolated populations are of particular interest since they may overcome some of the challenges in genetic investigations. Here we report the first genome-wide SNP-based study of the genetic structure in Xavante Indians.

Xavante is an indigenous population living in Mato Grosso state, Central Brazil. They comprise approximately 10,000 individuals, one of the largest indigenous groups in Brazil, and are Jê-speaking people [4], [5]. The earliest contact of Xavante with western culture was during the 18^th^ century in the Brazilian Central Plateau and this period was marked by epidemics, armed conflicts and forced labor imposed by the Portuguese colonial government. In the middle of 19^th^ century, in an attempt to escape mistreatment, they moved westward to their present habitat and remained relatively isolated until the early 20^th^ century. In the 1940s Brazilian government decided to stimulate settlements in its central region, considered a sparsely populated area, aiming to promote greater integration of this area with the rest of the country. As a result of this political decision the Xavante groups were forced to deal with the settlers and the post-contact period was characterized by epidemics and conflicts that resulted in a severe reduction of their population. By the end of the 1950s, the Xavante were reduced to small patches of settlements. However, in the last decades with the demarcation of their lands, health programs and establishment of peaceful contacts with non-Indians they experienced an increase in their population. Importantly, despite of the interaction with the outsiders, the Xavante maintain their own complex social organization and cultural values that were preserved over the years [5]–[7].

The aim of the present study was to characterize the genetic structure in Xavante Indians providing valuable baseline data for future genetic studies and to compare it with a Southeast Brazilian population [8], populations of the Human Genome Diversity Panel (HGDP-CEPH) [9], and populations of the HapMap Project, Phase III [10], which include individuals of Asian, African, European, and Mexican Ancestry.

## Material and Methods

### Population Samples

A cross-sectional study was conducted in the Sangradouro Reservation, Mato Grosso state, Brazil. The initial sample of Xavante Indians was comprised of 300 individuals and blood samples were collected from each subject. Genomic DNA was extracted from peripheral blood leukocytes using a commercial kit (Puregene DNA Isolation Kit, Gentra System, USA).

### Genotyping

Individuals were genotyped in 731,442 SNPs using Human Omni Express Bead Chip plataform (Illumina, San Diego, CA, USA). Genome-wide pair-wise identity-by-descent (IBD) estimated using the PLINK package [11] (http://pngu.mgh.harvard.edu/~purcell/plink/) confirmed the presence of related individuals in this sample. Genetic data were used for obtaining maximum likelihood estimates of relationship among pairs of individuals using the ML-Relate software [12] (http://www.montana.edu/kalinowski/Software/MLRelate.htm). This approach uses simulation to determine which relationships are consistent with the empirical genotype data, comparing putative relationships with possible alternatives. Only main pairs of individuals, such as parent-offspring, full-siblings, and half-siblings, are identified by the software. The ML-Relate software was unable to estimate the relationships using the complete panel of SNPs (731,442 SNPs), therefore we selected a set of approximately 10% independent markers (730 SNPs) to perform this analysis. The selection of unrelated individuals was conducted in several steps. The individual with the greatest number of relationships was identified and removed from the Xavante sample. Next, the relatedness was recalculated, generating other relationship structures. Again, the individual with the higher number of relatedness was removed and the relatedness was recalculated. This process was repeatedly made until we had only an unrelated sample. We identified 53 unrelated Xavante Indians in our sample.

Genotype data from the HapMap project (Phase 3) [10], the Human Genome Diversity Panel (HGDP-CEPH) [9], and from a Brazilian population sample were used to study the genetic structure of Xavante Indians. The HapMap Phase III data set is composed of 1,301 individuals of 11 populations that had been genotyped in almost 1.6 million genetic markers. The HGDP-CEPH database is composed of 1,068 individuals of 55 isolated populations. The Brazilian sample is constituted by 172 non-related individuals of a high degree of admixture [8], selected from residents in the municipality of São Paulo, the largest metropolitan area of the country. Genotyping for the Brazilian samples was performed using the Affymetrix SNP array 6.0 (Affymetrix, Santa Clara, CA, USA). All 11 panels of HapMap data set and 55 isolated populations of HGDP database were considered in our study. The PLINK package [11] was used for data management and quality control procedures on markers. Representative founder individuals were selected presenting a minimum percentage of 95% of high quality genotyped markers. Genetic markers were filtered using a maximum per-marker missing of 0.01 and a minor allele frequency (MAF) greater than 1%. The final dataset was composed of 1,198 HapMap, 940 HGDP, 172 São Paulo, and 53 Xavante individuals and 86,197 markers. Table 1 shows the populations and their respective number of individuals included in our study.

**Table 1:** Populations and number of individuals (N) included in the study.

Population	N
ASW (African ancestry in Southwest USA)	53
CEU (Utah residents with Northern and WesternEuropean ancestry from the CEPH collection)	112
CHB (Han Chinese in Beijing, China)	137
CHD (Chinese in Metropolitan Denver, Colorado)	109
GIH(Gujarati Indians in Houston, Texas, USA)	101
JPT (Japanese in Tokio, Japan)	113
LWK (Luhya in Webuye, Kenya)	110
MEX(Mexican ancestry in Los Angeles, California)	58
MKK (Maasai in Kinyawa, Kenya)	156
TSI(Toscans in Italy)	102
YRI (Yoruba in Ibadan, Nigeria)	147
Adygei	17
Balochi	24
BantuKenya	11
BantuSouthAfrica	8
Basque	24
Bedouin	46
BiakaPygmy	21
Brahui	25
Burusho	25
Cambodian	10
Colombian	7
Dai	10
Daur	9
Druze	42
French	28
Han	44
Hazara	22
Hezhen	8
Italian	12
Japanese	28
Kalash	23
Karitiana	14
Lahu	8
Makrani	25
Mandenka	22
Maya	21
MbutiPygmy	13
Melanesian	10
Miao	10
Mongola	10
Mozabite	29
Naxi	8
Orcadian	15
Oroqen	9
Palestinian	46
Papuan	17
Pathan	22
Pima	14
Russian	25
San	5
Sardinian	28
She	10
Sindhi	24
Surui	8
Tu	10
Tujia	10
Tuscan	8
Uygur	10
Xibo	9
Yakut	25
Yi	10
Yoruba	21
Xavante	53
São Paulo	172

The Indian leaders and the study participants were informed about the purposes of this study and gave their consent. The majority of the population gave their written consent, for the ones who were illiterate (14%), fingerprint impressions were used to document their approval. A Xavante health agent worked as an interpreter when necessary. This study was approved by Ethics Committee of Escola Paulista de Medicina, Universidade Federal de São Paulo and Brazilian National Ethics Commision (CONEP).

### Statistical Analysis

Principal component analysis (PCA) was applied to genotype data to infer continuous axes of genetic variation using the SmartPCA program of the Eigensoft package [13]. The axes of variation are defined as the top eigenvectors of a covariance matrix among samples and they are able to reduce the data to a small number of dimensions. PCA analysis were initially carried out using different subsets of populations to evaluate which populations were relevant to estimate the population structure of Xavante Indians. These datasets were composed by Xavante sample and, respectively: (a) other Amerindians samples; (b) Amerindians and Asians samples; (c) Amerindians, Asians and São Paulo samples; (d) the complete dataset. In these subsets, Han Chinese from Beijing, China (CHB), Chinese in Metropolitan Denver, Colorado (CHD), Gujarati Indians in Houston, Texas (GHI), and Japanese in Tokyo, Japan (JPT) (HapMap populations) and Cambodian represent the Asian ancestry; and Karitiana, Pima, Surui, and Maya (HGDP-CEPH) were used as representatives of the Amerindian ancestry. Mexican ancestry in Los Angeles, California (MEX HapMap populations) was also included in all analyzed subsets. MEX and São Paulo samples represent admixture populations. The three first principal components were plotted against each other and the different plots were analyzed. The São Paulo and Gujarati Indians in Houston, Texas, USA (GHI population of the HapMap project) samples have not contributed effectively to reveal the genetic population structure of Xavante samples and were removed from final dataset. Thus the final dataset for PCA was composed by Xavante Indians and all populations of HAPMAP and HGDP projects, excluding GHI and São Paulo samples. A PCA was then performed using this final dataset.

Pair-wise F_st_ estimates were also computed by SmartPCA program to characterize the genetic differentiation between Xavante Indians and the populations of the final dataset.

Next, we investigate the genome-wide genetic distance among individuals of all populations using a distance matrix computed by the PLINK package [11]. This matrix was obtained from complementary values of pair-wise identity-by-state. A neighbour-joining tree was constructed using PHYLIP (http://evolution.genetics.washington.edu/phylip.html) and visualized with HyperTree [14], a Java phylogenetic tree viewer (http://kinase.com/tools/HyperTree.html).

## Results

**Figure 1:**
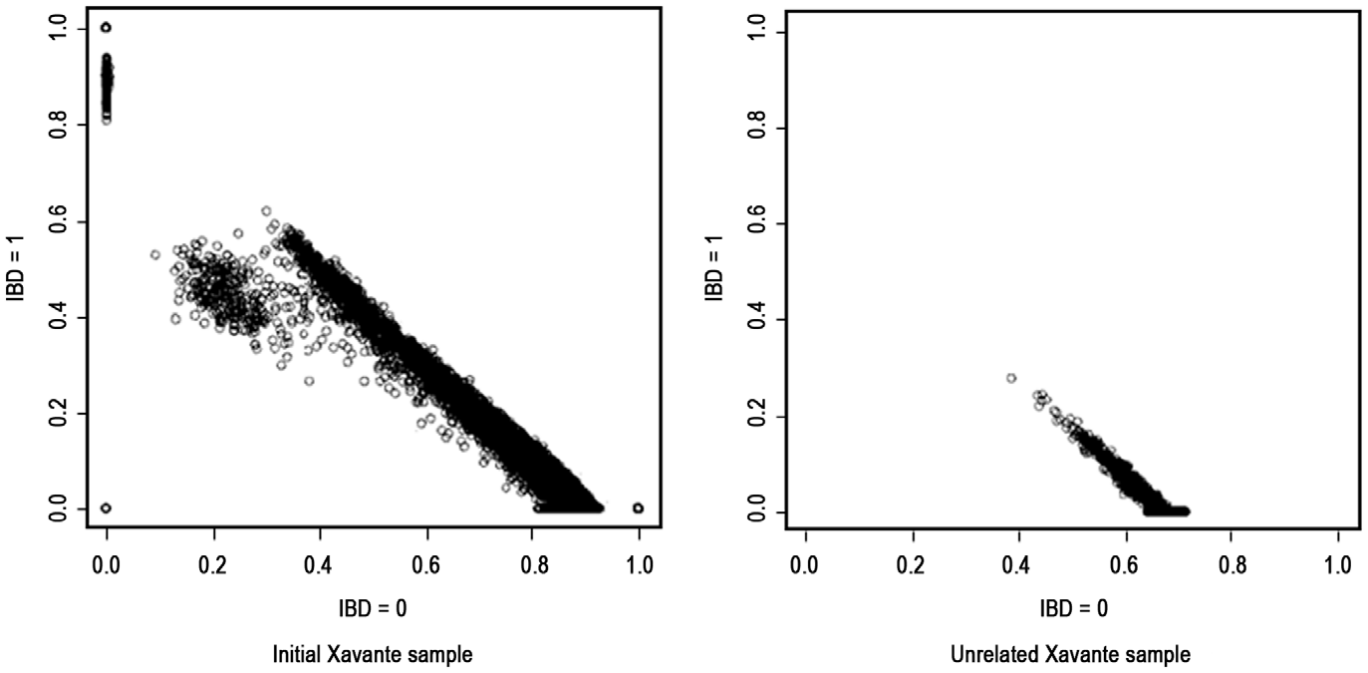
Probabilities that pairs of individuals, represented by circles, share zero (IBD = 0) vs. one (IBD = 1) allele identical-by-descent. Panel A represents the sharing of alleles among the initial sample of Xavantes, and panel B represents the sharing of alleles among unrelated individuals only.

We knew there was a familial structure in the Xavante Indians sample. Genealogical relationships can be represented mathematically as probabilities that individuals share zero, one, or two alleles identical-by-descent. All 731.442 markers were used to estimate the genome-wide pair-wise identity-by-descent using the PLINK package, confirming the presence of related individuals in our sample, as shown in Figure 1A (initial Xavante sample). In an unrelated sample, the pairs of individuals must be concentrated in the right-down portion of the graphic, showing a high probability of sharing zero alleles against a low probability of sharing one allele identical-by-descent. In our initial sample, the pairs are scattered in the graphic. The circle in the (0,0) position represents a duplicate sample, since there was no monozygotic twins in this population, which was used for QC measures.

The ML-Relate software was used to select unrelated individuals. Estimates of relatedness and relationship of main related pairs (parent-offspring, full-siblings, and half-siblings) are computed by maximum likelihood approach. After the removing process, a subset of 53 unrelated Xavante Indians were selected for the genetic population structure analysis. In Figure 1B, we show the sharing probability of alleles among pairs of individuals in this subset. ML-Relate has identified the main related pairs only, then the individuals more distantly related, as second- or third-degree related, remain in the sample. Indeed, the maximum probability of share one allele (IBD = 1) founded among unrelated individuals is 0.29, slightly greater than that observed between uncle/aunt and nephew/niece or among first cousins (0.25), confirming the presence of second-degree or more distant relationships in our final Xavante sample.

The group of 53 Xavante Indians was merged with HapMap, HGDP-CEPH, and São Paulo databases and the set of markers genotyped in all datasets was determined. The merged dataset is composed of 2,363 individuals (1,198 HapMap, 940 HGDP, 172 São Paulo, and 53 Xavante individuals) genotyped in 86,197 markers.

**Figure 2:**
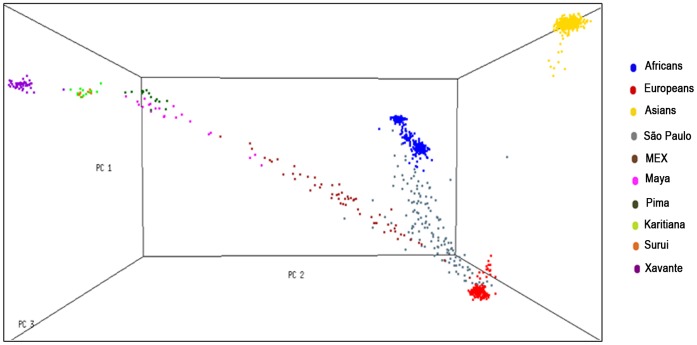
Three-dimensional plot of first three principal components (PC1, PC2, and PC3) computed from the merged dataset of populations. Yoruba, Maasai in Kinyawa, Kenia (MKK), African ancestry in Southwest, USA (ASW), and BantuKenya populations represent Africans; Utah residents with Northern and Western European ancestry from the CEPH collection (CEU), Adygei, and Basque populations represent Europeans; and Han Chinese in Beijing, China (CHB), Chinese in Metropolitan Denver, Colorado (CHD), Japanese in Tokyo, Japan (JPT), and Cambodian represent Asians.

Ten continuous axes of genetic variation (eigenvectors) were computed using the Eigensoft package. Principal component analyses (PCA) using different subsets of populations were firstly performed to investigate which populations were important for determine the genetic structure of the Xavante Indians. These datasets were composed by Xavante Indians and, respectively: (a) only Amerindians samples; (b) Amerindians and Asians samples; (c) Amerindians, Asians and São Paulo samples; (d) the complete dataset. Three-dimensional plots were provided to all analyses, considering the first three principal components. Comparing plots of each subset of populations, we decided to remove São Paulo and GHI samples from the final dataset for PCA, since these two populations do not enhance the determination of the eigenvectors of the Xavante sample. Thus the final dataset for PCA was composed by Xavante Indians and all populations of HAPMAP and HGDP projects, excluding only São Paulo and GHI samples. A principal component analysis was then performed using this final dataset. Although the São Paulo sample has been removed from our final dataset, we plotted the first three eigenvectors including it to illustrate the ancestry of the general Brazilian population, as shown in Figure 2. Xavante Indians (purple points), as expected, are concentrated in the Amerindian axis near the other populations of known Amerindian ancestry (Karitiana, Pima, Surui, and Maya). The São Paulo sample (black dots) is predominantly located between the European and African axis, showing a high degree of genetic admixture. This finding confirms early results [8], and it corroborates the long history of intermarriage between Europeans and Africans descent in the Brazilian population.

**Figure 3:**
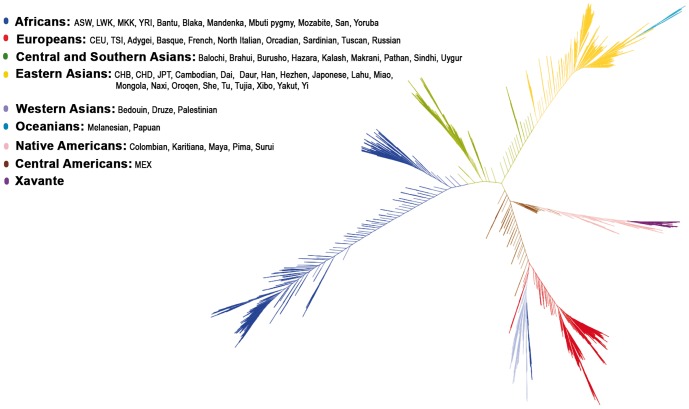
Neighbour-joining tree for the final dataset. The individuals were color labeled according to geographical distribution of their populations. ASW: African ancestry in Southwest USA; CEU: Utah residents with Northern and Western European ancestry from the CEPH collection; CHB: Han Chinese in Beijing, China; CHD: Chinese in Metropolitan Denver, Colorado; JPT: Japanese in Tokyo, Japan; LWK: Luhya in Webuye, Kenya; MEX: Mexican ancestry in Los Angeles, California; MKK: Maasai in Kinyawa, Kenya; TSI: Toscani in Italy; YRI: Yoruba in Ibadan, Nigeria.

We also studied the genetic similarity at the individual level from a genetic distance matrix obtained by calculating the complementary values of the genome-wide average proportion of alleles identical-by-state shared among pairs of individuals from all studied samples. The results of a neighbour-joining tree analysis are shown in Figure 3. The individuals were color labeled according to the geographical distribution of their populations. The Xavante Indians (in purple) clustered among the other Native American populations (in pink), as expected, corroborating the results obtained by the principal component analysis.

Pair-wise F_st_ estimates were also computed by the SmartPCA program of the Eigensoft package for all populations of our final dataset with more than 6 sampled individuals. The genetic differentiation between Xavante Indians and representative populations of the European, Asian, African, and Amerindian ancestry are shown in Table 2. We selected the HapMap populations CEU (Utah residents with Northern and Western European ancestry from the CEPH collection) to represent European ancestry, CHB, CHD, and JPT to represent Asian ancestry, and YRI (Yoruba in Ibadan, Nigeria) to characterize the African ancestry. Colombian, and Maya from HGDP-CEPH project characterized the Amerindian ancestry. Again, these results confirm some expected differences and resemblances among the set of studied populations. The genetic differentiation between Xavante Indians and populations of Ameridian, Asian, European, and African ancestry increased progressively.

**Table 2:** F_st_ estimates between Xavante Indians and representative populations of European (CEU), Asian (CHB, CHD, and JPT), African (YRI), and Amerindian (Colombian, and Maya) ancestry.

	Xavantes	CEU	CHB	CHD	JPT	YRI	Colombian	Maya
Xavantes (53)								
CEU (112)	0,186**							
CHB (137)	0,153**	0,109**						
CHD (109)	0,155**	0,111**	0,001					
JPT (113)	0,154**	0,112**	0,007	0,008**				
YRI (147)	0,271**	0,150**	0,182**	0,183**	0,184**			
Colombian (7)	0,075***	0,164**	0,133**	0,135**	0,133**	0,250**		
Maya (21)	0,058***	0,118**	0,095**	0,097***	0,096***	0,209**	0,087*	

* p<0.05;

** p<0.01;

*** p<0.001;

CEU: Utah residents with Northern and Western European ancestry from the CEPH collection; CHB: Han Chineses in Beijing, China; CHD: Chineses in Metropolitan Denver, Colorado; JPT: Japaneses in Tokyo, Japan; YRI: Yoruba in Ibadan, Nigeria. Numbers inside brackets represent the number of individuals in each population.

## Discussion

In this study we compared the genetic variation of 2,363 individuals (1,198 HapMap, 940 HGDP, 172 São Paulo, and 53 Xavante individuals) genotyped in 86,197 markers. Our results showed that the Xavante population has a low level of genetic admixture. This is consistent with the historical records of bottlenecks experience, cultural isolation with subsequent reduced gene flow. Despite using different methodologies, which difficult the comparison, our data are consistent with previous studies that have shown low level of admixture in the Xavante population suggesting that no significant changes in this villagés gene pool has occurred over last decades [5], [15]–[17].

Genetic studies of isolated populations have been subject of interest since they may help to map genes underlying simple monogenic, as well as, complex diseases. In isolated populations, monogenic disorders are less likely to show non-allelic heterogeneity [18]. The use of these populations in mapping complex disease have some advantages such as low genetic diversity, high degree of LD, restricted allelic and locus heterogeneity, reduced haplotype complexity and greater potential for identification of rare variants [19]–[21]. These benefits in association with cultural and environmental homogeneity make this population a good opportunity to identify novel susceptibility alleles for complex disease.

Principal component analyses demonstrate that the Xavante population is a distinct ethnic group more closely related to individuals of Amerindian ancestry and genetically distinct from other HapMap and HGDP populations and from São Paulo individuals.

By calculating pairwise F_st_ statistics, we found that the genetic differentiation between the Xavante population and representative populations of Amerindian, Asian, European and African ancestry increased progressively. These results are consistent with the Americas history of peopling that suggests a main colonization event from Siberia. The migration of humans from Eurasia to the Americas took place via Bering Strait and spread throughout North, Central and South Americas, diversifying into several culturally distinct native populations [22]–[27].

The findings from this study add to our understanding of genomic variation across the South American native populations and confirm that the Xavante is a closed Indian population that can provide a unique opportunity for genome-wide mapping studies of inherited disorders.
